# Socioeconomic factors and feeding in the first two years of life associated with molar incisor hypomineralisation

**DOI:** 10.3389/fdmed.2025.1573208

**Published:** 2025-06-18

**Authors:** Sara Ortega-Luengo, María Nieves Ábalos-Sanfrutos, María Isabel Calama-Fraile, Verónica Carballo-Tejeda, María Trinidad García-Vázquez, Antonia M. Caleya-Zambrano

**Affiliations:** ^1^ Madrid Health Service (SERMAS), Madrid, Spain; ^2^ Department of Dental Clinical Specialties, Faculty of Dentistry, Complutense University of Madrid, Madrid, Spain

**Keywords:** molar incisor hypomineralization, enamel, pediatric dentistry, molar incisor, dental defects, dental enamel hypoplasia

## Abstract

**Purpose:**

The main objective of this study was to evaluate the relationship between Molar Incisor Hypomineralisation (MIH) and socioeconomic factors in a sample of children in the Community of Madrid.

**Methods:**

An observational, descriptive, cross-sectional, and multicentre study was designed. After a previous calibration of all the examiners and following the diagnostic criteria of the European Academy of Paediatric Dentistry (EAPD), children between 8 and 16 years old were included from among the users of the dental services of eight primary care oral health units of the Madrid Health Service. The children underwent a dental examination, and the parents were asked to complete a questionnaire on possible socioeconomic factors related to the appearance of MIH. Factors such as sex, race, parental education, area of residence, exposure to tobacco and diet in the first two years of life were analysed. With MIH (yes/no) as the dependent variable, the *χ*2 test was used to compare categorical variables between MIH and non-MIH children. A logistic regression model was made with MIH (yes/no) as the dependent variable and the independent variables being those that were clinically relevant or significant in the bivariate analysis. Their associations are expressed as odds ratios (ORs).

**Results:**

Females were significantly more likely to have MIH. The prevalence of MIH decreased with age. Residents in Parla had MIH more often than residents of other municipalities of the Community of Madrid. No other socioeconomic factor studied was associated with MIH.

**Conclusions:**

Residents in the municipality of Parla had a higher frequency of MIH, which was a municipality with a low per capita income. When comparing the presence of MIH in breastfed children, statistically significant differences were observed. Logistic regression, however, did not suggest that breastfeeding could influence the presence of MIH. Further study is warranted on possible socioeconomic risk factors for MIH, such as household income, to lower the incidence of this pathology.

## Introduction

Molar Incisor Hypomineralisation (MIH) is an enamel alteration that was described for the first time in Sweden at the end of the 1970s, when paediatric dentists began to detect alterations in the dental enamel of the first molars and permanent incisors of unknown aetiology, not related to tooth decay (1). Although the term MIH was suggested in year 2000 by the European Academy of Paediatric Dentistry (EAPD) and was defined as “hypomineralisation of systemic origin, which affects between one and four permanent molars and is frequently associated with the involvement of the permanent incisors” (2), the problem of these defects in tooth enamel development has been known for over 100 years (3). Later, the term “molar hypomineralisation” was used to refer to cases in which there was no involvement of the incisors and only the first permanent molars were affected (4, 5), and “hypomineralisation primary second molars” referred to hypomineraliation present in the second primary molars (6).

MIH is a highly prevalent disorder. Worldwide, two recent revisions estimate it to be 13.1% and 14.2% (7, 8). In Spain, the prevalence of MIH is estimated to be 21.1% (8). Prevalence estimates in some areas include 21.8% in Valencia (9), 7.94% in Barcelona (10) or 28.63% in Madrid (11).

The aetiology of this enamel alteration is still completely unknown. The enamel becomes hypomineralised when there are alterations during the maturation phase of amelogenesis. Gene-mediated enamel mineralization is a sensitive process that can be altered by environmental factors (12). Although the aetiopathogenic mechanisms for MIH are unknown, it appears to be an acquired disease at some point in dental development rather than a genetic alteration (13). Nevertheless, recent research indicates that a genetic predisposition to MIH may exist in some individuals when certain environmental factors co-exist (14–17).

Possible ethiological factors related to enamel development defects are usually divided into three groups: prenatal, perinatal, and postnatal factors, according to the timing of their action (18). A large number of aetiological causes have been studied, including drug use, childhood illnesses, problems during pregnancy and maternal illnesses, environmental pollutants or contact with toxins (12, 19–32). Recently, several systematic reviews have been conducted with the objective of clarifying the possible causes of this alteration. However, it is not straightforward to obtain conclusive results, due to the fact that there are very few longitudinal studies available (33, 34).

Last years, some authors have proposed that there is a trend towards an increase in the number of cases of MIH (7). In the latest EAPD Clinical Guidelines 2022, epidemiological data show prevalence data between 2.9%–44% (17).In 2007 the prevalence of MIH in Madrid was estimated at 12.4% (35), though in a recently published study, the prevalence of MIH in this community was 28.63% (11). In light of the ﬁndings from previous studies, which were conducted in a similar population, and the absence of any other research in Madrid that has analysed the potential aetiological factors affecting individuals diagnosed with MIH, this study was designed to investigate whether socioeconomic and environmental factors might influence the aetiology of MIH.

## Material and methods

An observational, descriptive, cross-sectional, multicentre study was designed to determine which socioeconomic factors are related to the occurrence of MIH in boys and girls aged 8–16 years in different Oral Health Units (OHUs) of the Community of Madrid, Spain. This study was approved by the Ethics Committee for Drug Research of Hospital Clínico San Carlos (Internal Code: 21/162-E_Tesis).

### Study population and sample selection

The study population was boys and girls between the ages of 8 and 16 who attended the SERMAS oral health program and who met the inclusion criteria of the study: (1) Children who attended the OHUs during the study data collection period. (2) Children who had complete eruption of at least 1/3 of the occlusal surface of the first four permanent molars. (3) Children whose parents or guardians signed the informed consent form. Participation was voluntary. The following were excluded: (1) Children who presented any type of fixed attachment (tubes, bands, brackets, buttons, or attachments) that did not allow direct observation of the first permanent molars. (2) Children who were under who presented any medical, congenital, or acquired pathology related to the appearance of defects in enamel development. (3) Children who did not participate in the clinical inspection.

This publication is part of a research project carried out in the Public Health System of Madrid, where it first estimated the prevalence of MIH in a multicentre study (11). For the calculation of the sample size, EPIDAT 4.2 software was used. We assumed an incidence of MIH of 12%, according to the study by Comes (35) carried out in a population similar to ours. Setting a precision of 3% and a confidence level of 95%, we obtained a sample size of at least 451 children.

Given the interest in studying the possible relationship between some socioeconomic factors and the presence of MIH, we decided to select the OHUs according to the per capita income of the place where they were located. The number and location of all the SERMAS OHUs were determined by dividing them into two groups to facilitate randomization:

• Forty metropolitan OHUs located in Madrid city.
• Forty-six OHUs located in other municipalities of the Community of Madrid. Those located in towns with fewer than 20,000 inhabitants were considered rural OHUs.

The OHUs were ordered according to the per capita income of the place where they were located and were ordered from lowest to highest income, and the quartiles were calculated to select the OHUs that were in the first quartile and the fourth quartile. Those in the first quartile were classified as low income, and those in the fourth quartile were classified as high income.

We conducted two-stage sampling. In the first phase, by means of stratified random sampling, eight OHUs were selected—four from the metropolitan area and four from the rest of the municipalities—and in each of these groups, two from high-income areas and the other two from low-income areas. In the second phase, by means of consecutive sampling, 60 children were selected from each of the selected centres.

### Examiners: training and calibration

In each of the selected centres, a researcher was in charge of collecting dental data from each of the children who made up the sample. Before starting the data collection, the eight participating researchers participated in a training session on the EAPD diagnostic criteria for MIH. To ensure that all of them were calibrated, a questionnaire was designed with 29 photographs of teeth with different degrees of MIH. Each researcher answered the questionnaire twice. Once all the investigators had submitted their answers, Cohen's kappa was calculated for each of the dentists, and their answers were compared with the correct responses. The values obtained were between 0.74 and 0.94. This was an adequate degree of agreement, so the data collection phase started.

### Data collection

**Figure 1:**
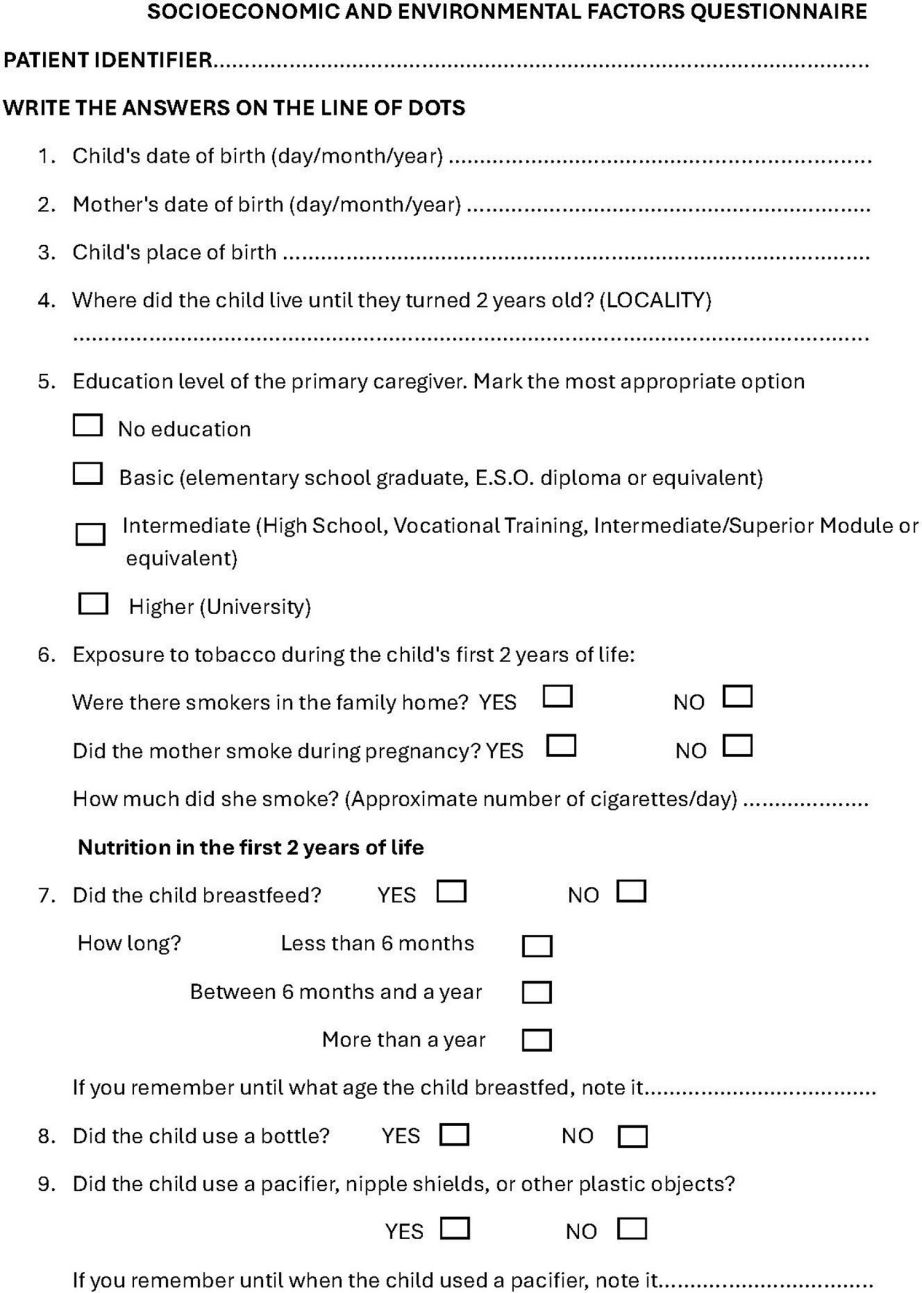
Questionnaire on socio-economic, environmental and nutritional factors during the first two years of life.

The data collection process consisted of two phases, an oral examination and a questionnaire with questions related to socioeconomic factors and habits of the children during their first two years of life that were completed by the parents. This questionnaire has not been validated but was developed by the researchers based on the available scientific evidence on socio-economic factors related to MIH (7, 12, 19, 23–32, 36) (Figure 1).

The oral examination was performed in the dental chairs of the OHU, from July 2022 to September 2023. Light from the dental equipment and examination mirror was used. Ball-ended probes were used to explore irregularities. If it was necessary to dry the mouth, gauze or cotton rolls were used. The diagnosis of MIH was made following the EAPD criteria (37).

### Statistical analysis

SPSS version 27 software was used to carry out the statistical analysis. The quantitative variables are described as mea*n* ± standard deviation. Categorical variables are described as *n* (%). With MIH (yes/no) as the dependent variable, the *χ*^2^ test was used to compare categorical variables between MIH and non-MIH children. A logistic regression model was made with MIH (yes/no) as the dependent variable and the independent variables being those that were clinically relevant or significant in the bivariate analysis. Their associations are expressed as odds ratios (ORs). Differences were considered statistically significant if *p* < 0.05.

## Results

The sample comprised 489 boys and girls between 8 and 16 years old. A total of 258 (52.76%) were girls and 231 were boys (47.23%). A total of 140 children were affected by MIH. Of those affected, 55 were boys (39.28%), and 85 were girls (60.71%).

Most of the participants (76.7%) were Caucasian, 44.6% of their parents had a higher education level or university equivalency, and 87.85% had resided in urban areas in their first years of life. The different socioeconomic characteristics of the children who participated in the study are shown in Table 1.

**Table 1:** Socioeconomic characteristics of the children in the sample.

Socioeconomic characteristics		MIH presence						
		No MIH			Yes MIH			*p*
		Media (DS)	Count	%	Media (DS)	Count	%	
Child's age at scanning (years)		11.19 (± 2,3)			10.57 (± 2,1)			**0**.**006**
Mother's age at child's birth (years)		31.95 (± 5,38)			31.39 (± 5,46)			0.74
Gender	Male		176	36.0%		55	11.2%	**0**.**026**
	Female		173	35.4%		85	17.4%	
Racial and ethnic characteristics	Caucasian		264	54.0%		111	22.7%	0.114
	Amerindian		49	10.0%		10	2.0%	
	Arab		19	3.9%		7	1.4%	
	Gypsy		10	2.0%		5	1.0%	
	Black		5	1.0%		3	0.6%	
	Asian		2	0.4%		4	0.8%	
Parentś level of education	No Education		6	1.2%		4	0.8%	0.81
	Basic		85	17.4%		33	6.7%	
	High School		97	19.8%		42	8.6%	
	Higher		158	32.3%		60	12.3%	
	No answer		3	0.6%		1	0.20%	
Income	High		185	37.8%		63	12.9%	0.10
	Low		164	33.5%		77	15.7%	
Place of residence (urban, rural and other)	Urban		304	62.2%		125	25.6%	0.416
	Other		26	5.3%		6	1.2%	
	Rural		19	3.9%		9	1.8%	

Bold values indicate *p* < 0.05.

The presence of MIH was analysed according to the location and income level of the health centre. When the presence of MIH was analysed in high- and low-income health centres, the *χ*^2^ test did not show statistically significant differences. The oral health units were in the municipalities of Madrid, Alcobendas, Parla and Algete.

**Figure 2:**
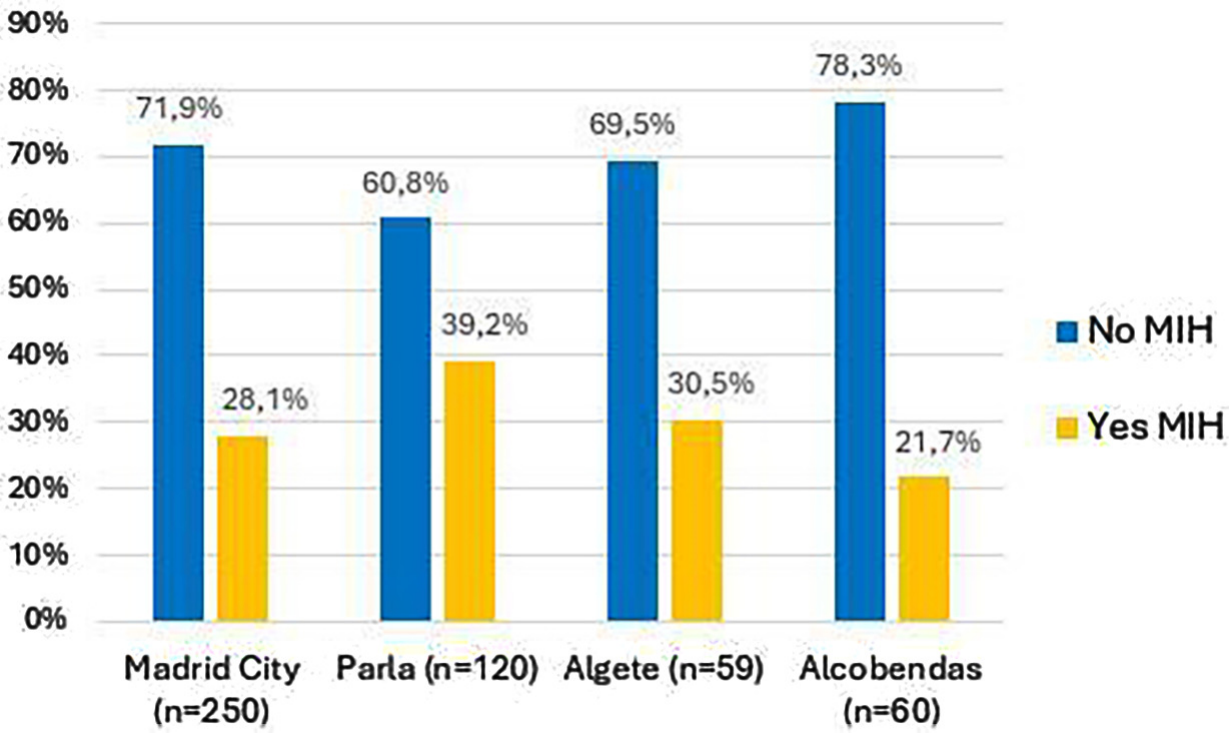
Bar chart comparing “No MIH” and “Yes MIH” percentages across four locations: Madrid City, Parla, Algete, and Alcobendas.

When the presence of MIH according to the municipality in which the OHU was located was studied (Figure 2), residing in the OHU of Parla, one of the low-income municipalities, was associated with having MIH (*χ*^2^ test, *p* = 0.003). No significant differences were found between other OHU locations in the rate of MIH.

We next analysed some environmental factors and eating habits that the participants in this study had during their first 2 years of life and their possible relationship with the appearance of MIH were analysed (Table 2). Breastfeeding was associated with a higher prevalence of MIH (*χ*^2^ test, *p* = 0.003). However, the duration of breastfeeding (less than 6 months, 6–12 months, or more than 12 months) was not associated with the presence of MIH. The other factors studied had no effect on the presence or absence of MIH.

**Table 2:** Environmental factors and eating habits during early childhood.

Environmental factors and eating habits		MIH presence				
		No MIH		Yes MIH		*p*
		Count	%	Count	%	
Contact with smoking environment	Non-smoker	256	52.60%	105	21.6%	0.84
	Family smoking	55	11.3%	22	4.5%	
	Mother smokes	36	7.4%	12	2.5%	
	No answer	2	0.4%	1	0.2%	
	Total	349	71.7%	140	28.8%	
Breastfeeding	Yes	292	59.8%	127	26.0%	**0**.**03**
	No	57	11.7%	12	2.5%	
	No answer	0	0.0%	1	0.2%	
	Total	349	71.5%	140	28.7%	
Baby bottle use	Yes	296	60.5%	109	22.3%	0.15
	No	51	10.4%	29	5.9%	
	No answer	2	0.4%	2	0.4%	
	Total	349	71.3%	140	28.6%	
Use of pacifiers and other plastic objects	Yes	259	53.0%	93	19.0%	0.20
	No	87	18.0%	46	9.4%	
	No answer	3	1.0%	1	0.2%	
	Total	349	72.0%	140	28.6%	

Bold values indicate *p* < 0.05.

To understand how each variable influenced the presence of MIH, logistic regression was carried out, in which the following variables were included: sex, age of the children at the time of the examination, age of the mothers at the birth of the children, location of the OHU, racial and ethnic characteristics, educational level of the main caregiver, residential area during the first 2 years of life, income in the area of location of the OHU, having had contact with smoking environments during the first 2 years of life, having breastfed, having used a bottle, and having had contact with other elements with plastic content. The results are shown in Table 3. Girls were significantly more likely than boys to have MIH (OR 1.546, CI 1.009–2.369) (*p* = 0.045); that is, the girls in this study had 1.54 times the likelihood of experiencing MIH than did the children. Older age at the time of the study made MIH less likely (OR 0.89, CI 0.814–0.942) (*p* = 0.019). In the rest of the variables studied, no significant results were found, so they do not seem to influence the presence of the disease.

**Table 3:** Logistic regression model (dependent variable presents MIH Yes/No).

Variables analyzed	Logistic regression					
	Reference Category	B	Sig.	Odds Ratio	95% C.I. OR	
					Lower	Upper
Girl	Boy	.436	.045	1.546	1.009	2.369
Age of child at scanning		−.112	.019	.894	.814	.982
Mother's age at child's birth		−.027	.243	.973	.930	1.018
Municipality where the OHUs is located	Madrid City		.318			
OHUs Parla		.640	.079	1.896	.929	3.872
OHUs Algete		.064	.892	1.067	.420	2.707
OHUs Alcobendas		−.188	.653	.829	.365	1.881
Racial and ethnic characteristics	Caucasian		.266			
Asian		1.172	.231	3.229	.474	21.995
Black		.254	.786	1.289	.207	8.024
Arab		−.545	.313	.580	.201	1.673
Amerindian		−.811	.078	.445	.180	1.095
Gypsy ethnicity		.003	.996	1.003	.249	4.045
Educational level	Higher education		.955			
No education		.393	.596	1.481	.346	6.329
Basic Education		−.002	.995	.998	.529	1.884
Medium studies		.055	.849	1.056	.602	1.852
Place of residence Urban/rural/other	Urban área of residence		.778			
Residence outside Spain		−.395	.505	.674	.211	2.149
Residence in rural areal		.109	.844	1.116	.376	3.313
Income level OHUs (High/low)	Low income	.041	.912	1.042	.505	2.149
Contact with smokers (Yes/No)	Yes	.150	.575	1.162	.688	1.960
Breastfeeding (Yes/No)	Yes	−.558	.134	.572	.276	1.187
Bottle use (Yes/no)	Yes	.088	.776	1.092	.597	1.994
Use of other plastic ítems (Yes/No)	Yes	.407	.138	1.502	.877	2.572

## Discussion

This study is part of a research project on MIH carried out in the Madrid public health system. In the first part, the prevalence and clinical characteristics of affected teeth were studied. Subsequently, we studied the presence of MIH and its association with environmental factors in patients who used public dental care, which Madrid provided through the Madrid Health Service (SERMAS). Few investigations have been carried out in the field of public health on MIH (38–40), and of these, only the one carried out by Haque Afzal has studied environmental factors (40). We analysed whether the age of the mother at the time the child was born could have any influence on the presence of MIH. On this topic, Balmer (19) suggested that this could influence the presence of MIH since pregnancies in older women tend to have more complications and show a greater association with the presence of MIH. In our study, no significant differences were found when studying this factor, in line with Ghanim (41). Although some authors have found differences between the presence of MIH and the ethnic origin of children (18, 28, 41, 42), we did not, in line with at least two other studies (16, 27, 35).

Other socioeconomic factors suggested in relation to MIH are the parents' level of education, family income level, or area of residence (23, 25–30). In the case of parental education, although Yi (28) related a higher maternal education with a lower incidence of MIH and Villanueva-Gutiérrez (30) associated a greater severity of MIH injuries with a lower educational level, since a low educational level is related to lower brushing frequencies, we did not find a link between the presence of MIH and the educational level of the parents. Moreover, we did not find differences between the presence of MIH and household income, unlike some studies that concluded that they do exist, such as that of Schwendicke (7). We believe that the differences observed between the presence of MIH and economic or educational level may be due to the association between income and cultural, since individuals with higher income and higher cultural level can more easily access dental care, so that preventive treatments could be carried out and try to maintain the integrity of the affected teeth, while in families with a lower income and lower cultural level, the difficulty in accessing dental treatments would favour the deterioration of the affected teeth before the disease is diagnosed. The fact that all the children in our study had access to oral care as a result of the existence of the Children's Dental Care Program (43), regardless of the income or cultural level of the parents, could explain why we did not find an influence of these variables. This feature of our research, which leads us to believe that families with a higher level of education could be underrepresented due to the easy access to private care and that the OHUs in high-income areas would go to people with fewer resources in the area. Dental examinations were carried out in the municipalities of Madrid Capital Area, Algete, Alcobendas and Parla.

When analysing the results of our sample, we observed that in the OHUs of the Madrid Capital area, there was a high prevalence of parents with university degrees, especially in high-income neighbourhoods. The above phenomenon was detected in Alcobendas, a municipality in northern Madrid. Although it is a municipality with a very high per capita (44) the majority of parents who attended the service recognized a basic level of education. In Parla, a municipality of more than 50,000 inhabitants in southern Madrid with the lowest income per capita, the most frequent parental educational level was medium, and in Algete, a municipality in northern Madrid with high income, the most frequent level of education was university. For this reason, although we studied users of a public health service, we believe the representation at the socioeconomic level was not skewed.

When analysing the presence of MIH in the children of the sample according to the municipality where the OHUs was located, we found statistically significant differences between the children of the municipality of Parla and those of the other municipalities. The children of Parla presented MIH at a rate of 39.2%, almost double that of the children of Alcobendas, who were the ones who presented the lowest rate of MIH in this study (21.7%). The municipality of Parla is one of the municipalities with the lowest income per capita in the Community of Madrid, while Alcobendas has one of the highest incomes. By also relating the level of education of the parents of the children in these municipalities, we observed that, in Parla, many of parents had higher education, while in Alcobendas, they reported basic-level studies. We believe that the differences found in the children of Parla may be due to this disparity in educational level, since parents with a higher education level may demand more health care and therefore bring their children to a higher proportion of dental check-ups at the OHU than those with a lower education level. It would be desirable for health officials to encourage oral health programs for families with fewer resources since they may be unaware of their existence. On the other hand, the sample of the municipality of Alcobendas may be underrepresented, and parents with higher educational levels with children affected by MIH do not use public health and go directly to private dentists, so these children will not have been counted. This would mean we found a lower rate of MIH in Alcobendas not because it is rarer there but because they do not attend public consultations and were not included in the study.

Drugs, early childhood diseases, and environmental pollutants have been identified as possible causes of MIH due to their influence on the mineralization of dental enamel (12, 21–24). Some authors suggest that living in urban areas may favour the diagnosis of MIH because of the ease of access to dental consultations and medical treatment (22, 23), although other researchers have found a higher prevalence of MIH in rural areas (24). In our study, we did not find a significant effect of the urban or rural environment, though the sample of children who lived in rural areas in the first 2 years of life was small. Geographical distribution of the population in Madrid shows a concentration in urban areas, with a smaller proportion of inhabitants in rural zones. This can affect the ability to recruit a representative sample of rural patients, as most participants come from urban areas. It is recommended that future studies consider strategies to increase the representation of rural patients, such as collaborating with health centres in small towns or implementing more inclusive sampling methods.

In addition, we studied the associations between MIH and exposure to tobacco, plastic elements, and breastfeeding (due to the possible presence of toxins in breast milk) in the children in our sample since these factors are related to the aetiological mechanism of MIH (12, 31–33). Our findings agree with others (23, 38, 45, 46) in that there was no significant effect of any of those variables. Non-halogenated polycyclic aromatic hydrocarbons (PAHs) present in tobacco have been described as a possible etiological mechanism of MIH (31). Garot (33), in a systematic review, found no differences in the presence of MIH in children who had been exposed to smokers during their early years of life. Ilczuk-Rypula (45) in Poland and Wuollet (23) in Finland also found no differences in their studies. Lim, in a study conducted in the United Kingdom (46), was unable to find firm evidence of an association between MIH and maternal smoking habits. Our sample was not influenced by contact with smokers, whether the smoker was the mother or a partner or the number of cigarettes smoked, unlike Lee (47), who reported an OR of 2.37 for MIH in children whose mothers smoked during pregnancy. The presence of dioxins in breast milk has been suggested to be a factor associated with the appearance of MIH (31, 48). The existing studies on this topic are controversial. While the studies by Koruyucu (49) and Ghanim (41) revealed significant differences in the presence of MIH as a function of breastfeeding time, other researchers have not found this (7, 50–52).

In our study, statistically significant differences were found in the presence of MIH between children who breastfed and those who did not (*χ*^2^ test, *p* = 0.026), but the logistic regression analysis, which included breastfeeding and other confounding factors, did not reveal statistically significant differences between being breastfed or not and the presence of MIH. We also did not find a relationship between the duration of breastfeeding and the presence of MIH.

Jedeon (32) suggested in different publications that endocrine disruptors, such as bisphenol A (BPA), can interfere with enamel mineralization, favouring the appearance of MIH (32, 33). BPA is present in food containers and other utensils for babies and young children (53, 54). Several investigations have studied the relationship between the use of bottles and the presence of MIH (9, 55), without finding statistically significant effects.

Elzein (55), in Lebanon, studied the relationship between MIH and its possible association with various pre- and post-natal factors and found no difference in the presence of MIH in children who breastfed for more than 6 months compared to those who did not. However, he did detect differences in children breastfed by mothers who consumed canned foods, linking it to the possible transfer of bisphenol A from the canned foods through breast milk. In our study, we did not find an effect of feeding from a bottle or other plastic devices on the presence of MIH.

Our study has some limitations, such as memory bias. We also considered sample representativeness as a potential limitation, that could influence the external validity of our results. In this study, all participants were users of the public health system. Predominantly, dentistry in the Community of Madrid has been provided privately, so the studied population may not be entirely representative. Often, public health patients are those with fewer economic resources and/or more health issues. To counteract this, medical centres from areas with both high and low income levels were selected. We cannot rule out the possibility of confounding bias when analysing the influence of socioeconomic variables on the presence of MIH. Another possible limitation is that we used a global income indicator of the municipality or the neighbourhood instead of asking about the individual household income because we have found, in our previous experiences, that these questions tend to cause rejection and are not answered truthfully. Perhaps the ideal would have been to ask each family unit about their income level individually. On the other hand, the fact that patients were users of the public health system can also be seen as a strength of this research, as the ethnic and racial variability, as well as the socioeconomic status of the participants, is likely to have been greater than if the sample had been obtained from private clinics. Another limitation of the present study is the lack of validation of the questionnaire passed on to the parents of the patients, although it is based on the available scientific evidence and the literature found. We consider it interesting to carry out future studies to validate these questionnaires.

## Conclusions

Among the users of public dentistry services in Madrid, girls were more likely than boys to have MIH. The prevalence of MIH decreased with increasing age of the children in the study. Statistically significant differences were observed when comparing the presence or absence of MIH in children living in Parla and children who had been breastfed. Given the results found in this municipality, we believe that in future research, it would be beneficial to factor in the individual (household) income level of the participants.

When comparing the presence of MIH in breastfed children, statistically significant differences were observed. Logistic regression, however, did not suggest that breastfeeding could influence the presence of MIH. No other socioeconomic factor studied was associated with MIH.

We believe it is very important to continue studying possible etiological factors in order to prevent this dental alteration that affects the quality of life of pediatric patients.

## Data Availability

The raw data supporting the conclusions of this article will be made available by the authors, without undue reservation.
